# Brain Health Promotion in the LMIC Context: Innovative Approach of the Africa‐FINGERS Program

**DOI:** 10.1002/alz70860_098552

**Published:** 2025-12-23

**Authors:** Chinedu T Udeh‐Momoh, Miia Kivipelto, Investigators of The Africa‐FINGERS Program

**Affiliations:** ^1^ Division of Public Health Sciences, Wake Forest University School of Medicine, Winston‐Salem, NC, USA; ^2^ Brain and Mind Institute, Aga Khan University, Nairobi, Kenya; ^3^ FINGERS Brain Health Institute, Stockholm, Sweden; ^4^ Division of Clinical Geriatrics, Center for Alzheimer Research, Department of Neurobiology, Care Sciences and Society, Karolinska Institutet, Stockholm, Sweden; ^5^ The Ageing Epidemiology (AGE) Research Unit, School of Public Health, Imperial College London, London, United Kingdom

## Abstract

**Background:**

Dementia is a growing public health crisis, with over 60% of cases projected to occur in low‐ and middle‐income countries (LMICs) by 2050. Africa is expected to experience the fastest increase in prevalence, yet research on culturally tailored risk reduction strategies remains limited. Africa‐FINGERS is the first coordinated initiative implementing contextually adapted multimodal dementia prevention across multiple African nations. This initiative integrates validated dementia risk scores, biomarker‐informed detection, and community‐based system dynamics approaches to address region‐specific risk factors.

**Methods:**

Africa‐FINGERS builds upon the World‐Wide FINGERS (WW‐FINGERS) model, adapting it to Africa's diverse cultural, socio‐economic, and healthcare landscapes. The program harmonizes dementia risk prediction using established risk scores validated in African cohorts. Biomarker‐driven detection incorporates fluid‐based (CSF, blood, saliva) and neuroimaging markers, coupled with a multidimensional lifestyle intervention tailored to contextual factors identified through ethnographic deep cultural phenotyping. These factors include multidimensional poverty, social connectedness, and malnutrition. Interventions integrate financial support, mindfulness, and culturally informed cognitive enrichment practices. Recruitment pipelines leverage existing epidemiological cohorts, ensuring rapid enrollment of at‐risk individuals. The intervention will be evaluated for acceptability, feasibility, and adherence to support sustainable integration into African health systems.

**Results:**

Preliminary findings from external validation of dementia risk scores in African cohorts demonstrate high concordance with Western‐derived models, with necessary modifications to account for Africa‐specific risk signatures. Risk factor analyses from the Ibadan Nigeria Dementia Study, AD‐Detect Kenya, and Brain Resilience Kenya highlight key predictors, including hypertension, stroke history, low social engagement, and metabolic risk factors, underscoring the relevance of targeting these modifiable domains. Community‐based participatory research work to be presented suggests strong acceptability and feasibility of multimodal interventions incorporating local values, social support systems, and economic empowerment strategies.

**Conclusion:**

Africa‐FINGERS pioneers precision dementia prevention in Africa by integrating biomarker‐informed risk detection with culturally adapted multimodal interventions. This approach bridges global research with local implementation, enabling comparative analyses with WW‐FINGERS studies and informing risk reduction strategies for Black Africans in Africa and the diaspora. Aligning with WW‐FINGERS and leveraging Africa's unique epidemiological and cultural landscape, Africa‐FINGERS serves as a scalable and replicable model for dementia prevention in low resource settings.

